# Predictive biomarkers for dasatinib treatment in melanoma

**DOI:** 10.18632/oncoscience.20

**Published:** 2014-03-12

**Authors:** Alex J. Eustace, Susan Kennedy, Anne-Marie Larkin, Thamir Mahgoub, Dimitrios Tryfonopoulos, Lorraine O'Driscoll, Martin Clynes, John Crown, Norma O'Donovan

**Affiliations:** ^1^ National Institute for Cellular Biotechnology, Dublin City University, Dublin 9, Ireland; ^2^ Research Foundation, Royal Victoria Eye and Ear Foundation, Royal Victoria Eye and Ear Hospital; ^3^ Department of Medical Oncology, St Vincent’s University Hospital, Dublin 4, Ireland; ^4^ School of Pharmacy & Pharmaceutical Sciences, Trinity College Dublin, Dublin 2, Ireland.

**Keywords:** Melanoma, dasatinib, biomarker, ANXA1, CAV-1, EphA2

## Abstract

Dasatinib has anti-proliferative and anti-invasive effects in melanoma cell lines. However clinical trials have shown modest activity for dasatinib in metastatic melanoma. Although dasatinib targets SRC kinase, neither expression nor phosphorylation of SRC appears to predict response to dasatinib. Identification of predictive biomarkers for dasatinib may facilitate selection of melanoma patients who are more likely to respond to dasatinib. We correlated the anti-proliferative effects of dasatinib in 8 melanoma cell lines with expression of a previously identified 6-gene biomarker panel. We examined the relationship between response to dasatinib and expression of each gene at both the mRNA and protein level. Dasatinib inhibited growth in 3 of the 8 cell lines tested. mRNA expression of the panel of 6 biomarkers did not correlate with response, whilst elevated protein expression of ANXA1, CAV-1 and EphA2 correlated significantly with response to dasatinib in the panel of cell lines. Expression of ANXA1, CAV-1 and EphA2 were analysed in 124 melanoma samples by immunohistochemistry. ANXA1 protein was detected in 81 % (97/120) of tumours, CAV-1 in 44 % (54/122) of tumours and EphA2 in 74 % (90/121) of tumours. Thirty one % (35/113) of tumours tested expressed all three markers and 19 % (21/112) had moderate or strong expression of ANXA1, CAV-1 and EphA2. Seventeen percent (19/112) of melanoma samples were positive for SRC kinase expression, combined with high expression of ANXA1, CAV-1 and EphA2. This subgroup may represent a population of melanoma patients who would be more likely to derive clinical benefit from dasatinib treatment.

## INTRODUCTION

SRC kinases, a family of structurally related non-receptor tyrosine kinases, have been implicated in cell proliferation and migration/invasion in preclinical models of melanoma [1-3]. Members of the SRC family, including c-SRC and Yes, are expressed in melanoma cells [1, 4, 5] and expression of c-SRC is elevated in melanoma cells compared to normal melanocytes [1, 4, 5]. In a study of 35 melanomas, phospho-SRC (p-SRC) was detected in approximately 50 % (17/35) by immunohistochemical analysis [6].

Previous studies have linked SRC inhibition with the control of proliferation in melanoma cells [6, 7] and SRC inhibition induces apoptosis in melanoma cells [7]. Dasatinib, a multi-target tyrosine kinase inhibitor which targets BCR-Abl, Src kinases, c-KIT, PDGFR and ephrin-A receptor kinases, is currently a first line treatment in chronic myeloid leukaemia. Dasatinib inhibits proliferation and invasion in melanoma cell lines [7]. However, initial clinical trials of dasatinib have been disappointing in non c-Kit mutated metastatic melanoma [8].

It is widely recognized that the success of targeted therapies depends on identification of appropriate predictive biomarkers, such as HER2 for trastuzumab treatment in breast cancer and Bcr-Abl for imatinib or dasatinib in chronic myeloid leukaemia treatment [9, 10]. Consistent with our previous results [7], Jilaveanu *et al* (2011) found that neither expression nor phosphorylation of SRC correlated with response to dasatinib in a panel of melanoma cell lines [5], therefore identifying the need for novel biomarkers of response to dasatinib.

Huang *et al* [11] previously correlated microarray data and sensitivity to dasatinib in 23 breast cancer cell lines and identified a panel of six genes that predict response to dasatinib. Five of these genes, Annexin-A1 (ANXA1), Caveolin-1 (CAV-1), Caveolin-2 (CAV-2), Ephrin-A2 (EphA2) and PTRF, are expressed at higher levels and one gene, Insulin Growth Factor Binding Protein 2 (IGFBP2), is expressed at lower levels in dasatinib-sensitive cell lines compared to dasatinib-resistant cell lines. A number of other studies have also tested potential biomarkers of response to dasatinib *in vitro*. In breast cancer cell lines, elevated expression of CAV-1, moesin and yes associated protein-1 (YAP- 1) predicted sensitivity to dasatinib [12, 13]. Elevated expression of androgen receptor, prostate specific antigen, cytokeratin 5, urokinase-type plasminogen activator and EphA2 correlated with dasatinib sensitivity in prostate cancer cell lines [14]. Finally, in ovarian cancer cell lines elevated expression of CAV-1, ANXA1 and EphA2 correlated with sensitivity to dasatinib [15].

We have tested the 6-gene biomarker panel identified by Huang *et al* [11] in a panel of melanoma cell lines and assessed the frequency of expression of 3 potential dasatinib predictive biomarkers in melanoma specimens.

## RESULTS

### Sensitivity to dasatinib

**Figure 1:**
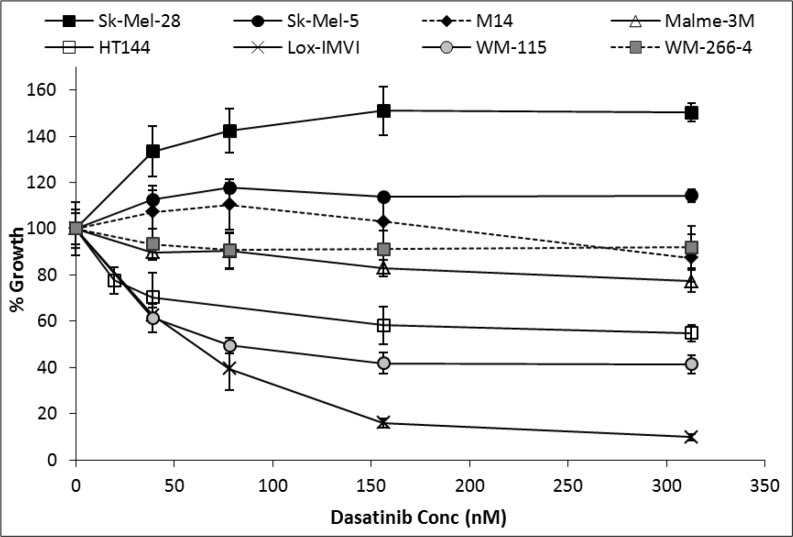
Percentage growth inhibition by dasatinib treatment for 5 days in a panel of melanoma cell lines

Sensitivity to growth inhibition by dasatinib varied across the panel of melanoma cell lines tested (Figure 1). Lox-IMVI and WM-115 displayed the greatest sensitivity to dasatinib with IC_50_ values of 35.4 nM (± 8.8 nM) and 79.3 nM (± 11.7 nM), respectively, whilst HT144 displayed maximum growth inhibition of 45 % when treated with 310 nM dasatinib. Malme-3M displayed limited sensitivity to dasatinib with 25 % growth inhibition at 310 nM. Dasatinib showed minimal inhibition of growth of WM266-4 and M14 cells, 8 % and 13 % respectively. Dasatinib treatment increased the growth of Sk-Mel-28 and Sk-Mel-5 cells. Melanoma cell lines which showed greater than 30% growth inhibition with 155 nM dasatinib were defined as sensitive (Lox-IMVI, WM-115 and HT144).

Error bars represent the standard deviation of triplicate experiments.

### RNA expression of biomarker panel in melanoma cell lines

**Figure 2:**
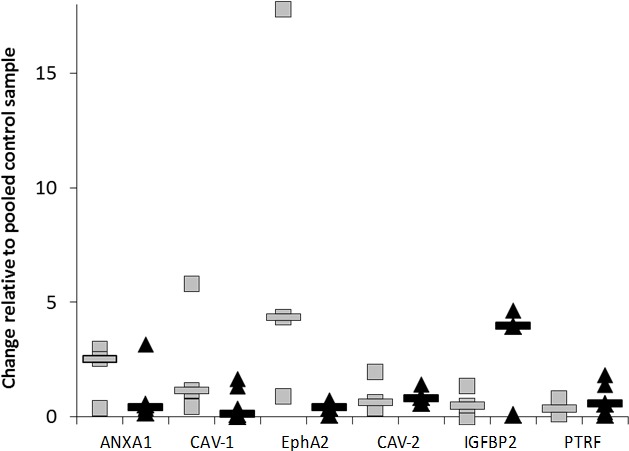
mRNA expression levels of ANXA1, CAV-1, EphA2, CAV-2, IGFBP2 and PTRF candidate markers measured by q-RT-PCR

qRT-PCR analysis was performed on the 3 dasatinib sensitive versus the 5 dasatinib resistant melanoma cell lines, to determine the mRNA levels of the 6 gene biomarker panel (Figure 2 and Supplementary Figure 1). Cell lines were individually compared to a control sample (a pooled sample which consisted of an equal volume of mRNA from each of the cell lines) which reflects the average mRNA expression of all the cell lines tested. No significant differences were observed in ANXA-1, CAV-1, CAV-2, EphA2, IGFBP2 or PTRF mRNA levels between dasatinib sensitive and dasatinib resistant cell lines.

Grey Boxes represent the fold change in expression of markers for dasatinib sensitive cell lines whilst black triangles represent the fold change in expression of markers for dasatinib resistant cell lines. ‘—’ indicates the median result for either the dasatinib sensitive or dasatinib resistant cell lines for each candidate gene.

### Protein expression of biomarker panel in the melanoma cell lines

**Figure 3A:**
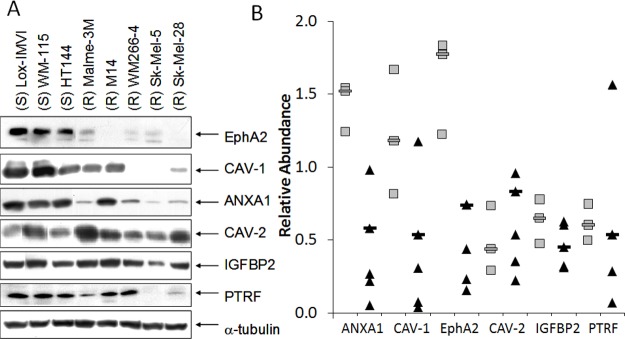
Immunoblotting for EphA2, CAV-1, ANXA1, CAV-2, IGFBP2 and PTRF in dasatinib sensitive (S) and dasatinib resistant (R) melanoma cell lines. **3B:** Expression levels of ANXA1, CAV-1, EphA2, CAV-2, IGFBP2 and PTRF candidate markers measured by densitometry from triplicate western blots. Grey squares represent the average expression of triplicate analysis of individual dasatinib sensitive cell lines compared to the expression in the dasatinib resistant cell lines represented by black squares. ‘—’ indicates that the median result of either dasatinib sensitive or dasatinib resistant cell lines.

Western blot analysis was performed for each of the proteins encoded by the 6-gene predictive biomarker panel (Figure 3 and Supplementary Figure 2). ANXA-1 was detected in all cell lines; but significantly higher levels were detected in dasatinib sensitive cell lines (p = 0.04). CAV-1 was detected in all of the sensitive cell lines but in only 3 of the 5 resistant cell lines. CAV-1 expression levels were significantly higher in dasatinib sensitive cell lines (p = 0.05). EphA2 was detected in 6 of the 8 melanoma cell lines tested. Significantly higher levels of EphA2 were detected in dasatinib responsive cell lines compared to dasatinib resistant cell lines (p = 0.02). Using the median value as a cut-off we categorised each of the cell lines as high or low expressors for each of the markers. Again high expression of either ANXA1, CAV-1 or EphA2 predicted sensitivity to dasatinib in the cell line panel, with EphA2 yielding the strongest p value (p=0.028, p=0.028, p=0.005, respectively). Combined analysis of the markers suggests that measuring ANXA1 and EphA2 or CAV-1 and EphA2 has similar predictive power as measuring all 3 markers (p=0.018) (Supplementary table 1). No significant difference in expression of CAV-2, IGFBP2 or PTRF was detected between dasatinib responsive and resistant cell lines.

Pearson correlation coefficient analysis was used to examine the relationship between mRNA and protein expression of ANXA1, CAV-1, CAV-2, EphA2, IGFBP2 and PTRF in the panel of melanoma cell lines (Supplementary table 2). A significant positive correlation was observed between ANXA1 (r = 0.731; p = 0.039), CAV-1 (r = 0.810; p = 0.015) and PTRF (r = 0.731; p = 0.039) mRNA and protein expression, whilst a correlation was observed for EphA2 (r = 0.694; p = 0.056) but did not achieve statistical significance. Expression of mRNA and protein did not correlate for CAV-2 or IGFBP2. There was also a significant positive correlation between protein expression of ANXA1, CAV-1 and EphA2, whereby high expression of the individual protein markers was associated with high expression of the other markers (ANXA1 vs CAV-1 p = 0.013; ANXA1 vs EphA2 p = 0.0001; CAV-1 vs EphA2 p = 0.003).

### Biomarker expression in human melanoma samples

**Figure 4:**
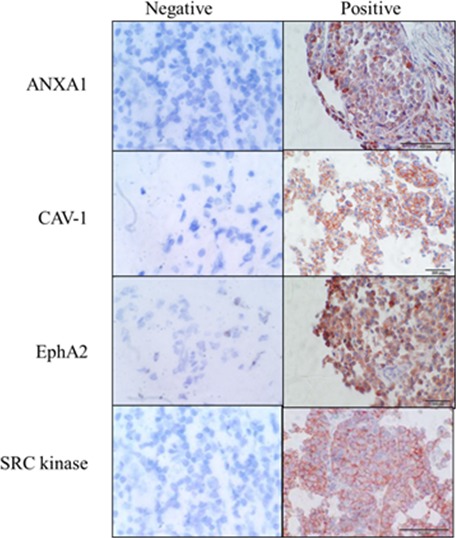
Representative immunohistochemical staining of melanoma samples showing ANXA1, CAV-1, EphA2 and SRC kinase positive staining

We examined expression of SRC kinase and the 3 potential predictive biomarkers, ANXA1, CAV-1 and EphA2, in a cohort of 125 melanoma specimens (Figure 4, Supplementary table 3).

Negative controls represent melanoma samples where no staining is observed in tumour cells. Scale bar - 200 μM, original magnification – 200X. All sections were counterstained with haematoxylin.

SRC is one of the kinases targeted by dasatinib. We and others have shown that levels of SRC expression or phosphorylation do not predict sensitivity to dasatinib in melanoma cell lines [5, 7]. However, all of the cell lines we tested were positive for SRC expression. Therefore, it is possible that SRC expression is a prerequisite for dasatinib sensitivity.

SRC kinase was expressed in 94/122 (77 %) of melanoma samples with 66/82 (80 %) of primary and 28/40 (70 %) of metastatic samples positive for SRC kinase (Table 1). A greater percentage of tumours with Clark’s levels of 4 or 5 showed higher intensity staining (3+: 40.7%) for SRC kinase staining than tumours with Clark’s level lower than 4 (3+: 30.5%) (p = 0.024, Chi- Square test, Supplementary table 4).

**Table 1:** Frequency and intensity of expression of ANXA1, CAV-1, EphA2 and SRC kinase in melanoma samples as measured by immunohistochemistry

IHC Results	ANXA1	CAV-1	EphA2	SRC kinase	Positive for 4 markers	SRC positive tumours with moderate/strong staining for ANXA1, CAV-1, and EphA2
All Samples	97/120 (81 %)	54/122 (44 %)	90/121 (74 %)	94/122 (74 %)	35/113 (31 %)	19/113 (17%)
Primary	67/81 (83 %)	39/82 (48 %)	68/82 (83 %)	66/82 (80 %)	25/77 (32 %)	14/77 (18 %)
Negative	14/81 (17%)	43/82 (52 %)	14/82 (17 %)	16/82 (20 %)		
Weak	5/81 (6 %)	10/82 (12 %)	14/82 (17 %)	15/82 (18 %)		
Moderate/Strong	62/81 (77 %)	30/82 (37 %)	54/82 (66 %)	51/82 (62 %)		
Metastatic	30/39 (77 %)	15/40 (38 %)	22/39 (56 %)	28/40 (70 %)	10/36 (28 %)	5/36 (14 %)
Negative	9/39 (23 %)	25/40 (62 %)	16/39 (41 %)	12/40 (30 %)		
Weak	2/39 (5 %)	1/40 (3 %)	2/39 (5 %)	7/40 (18 %)		
Moderate/Strong	28 39 (72 %)	14/40 (35 %)	21/39 (54 %)	21/40 (53 %)		

ANXA1 was expressed in 97/120 (81 %) of the melanoma samples (Table 1), with 67/81 (83 %) of primary and 30/39 (77 %) of metastatic melanoma samples positive. The intensity of ANXA1 staining was higher in patients with a lower Breslow thickness compared to those with higher Breslow thickness (p = 0.047; Kruskal-Wallis test) (Supplementary table 5). However, a statistically significant association was not observed when Breslow thickness was divided into categories.

CAV-1 was expressed in 54/122 (44 %) of melanoma samples, 39/82 (48 %) of primary and 15/40 (38 %) of metastatic melanoma samples (Table 1). CAV-1 expression was inversely associated with age, whereby patients who were younger than 60 years were more frequently positive (70.4 %) for CAV-1 than patients greater than 60 years old (36.4 %) (p=0.004) (Supplementary table 6).

EphA2 was expressed in 90/121 (74 %) of melanoma samples, and was detected more frequently in in primary samples 68/82 (83 %) compared to metastatic samples 22/39 (56 %) (p = 0.003; Chi-squared test) (Table 1). EphA2 expression did not correlate with the clinicopathological parameters (Supplementary table 7).

Co-expression of SRC, ANXA1, CAV-1 and EphA2 was detected in 35/113 (31 %) of melanoma samples (Table 1). 25/77 (32 %) of primary tumours and 10/36 (28%) of metastatic samples were positive for expression of all four markers.

Analysis of ANXA1, CAV-1 and EphA2 in the cell lines suggests that their combined elevated expression may predict response to dasatinib. Therefore, we also examined the number of samples with moderate or strong staining for ANXA1, CAV-1 and EphA2 as determined by IHC. 90/120 (75 %) of samples had moderate/strong staining for ANXA1, 44/122 (36 %) had moderate/strong staining or CAV-1, 75/121 (62 %) had moderate/strong staining for EphA2 (Table 1). Moderate/strong expression of all three markers (ANXA1, CAV-1 and EphA2) was found in 21/113 (19 %) of the melanoma samples, 16/77 (21 %) of primary melanoma samples and 5/36 (14 %) of metastatic samples. If SRC kinase staining is added to the panel, 19/113 (17 %) of the melanomas express detectable levels of SRC kinase and have moderate/strong expression of ANXA1, CAV-1 and EphA2 (Table 1).

## DISCUSSION

Recent advances in the development of therapeutic antibodies targeting immune checkpoint modulation and BRaf/Mek kinase inhibitors have dramatically improved treatment options and prognosis for patients with metastatic melanoma [16, 17]. However, not all melanoma patients respond to these therapies and in the case of BRaf inhibitors, the development of acquired resistance has been associated with alternative signalling pathways, including SRC kinase signalling [18]. Thus targeting SRC kinase may be beneficial in some melanoma patients. The challenge is to identify the melanoma patients that will benefit from SRC kinase inhibition. In this study we aimed to address this challenge by identifying predictive biomarkers of response to dasatinib treatment.

In a previous study in breast cancer cell lines, sensitivity to dasatinib was defined as at least 60 % inhibition of cell proliferation with 1 μM dasatinib, moderate sensitivity as 40-59 % inhibition and resistance as less than 40 % inhibition (based on the assumption that concentrations higher than 1 μM would not be achievable *in vivo*) [12]. In our study of melanoma cell lines, we have classified cell lines which displayed greater than 30 % inhibition of proliferation at 155 nM as dasatinib responsive (Lox-IMVI, WM-115 and HT144) and cell lines with less than 30 % inhibition of proliferation at 155 nM as dasatinib resistant (Malme-3M, M14, WM-266-4, Sk-Mel-5 and Sk-Mel-28). The concentration of 155 nM dasatinib was selected to represent a concentration close to the median peak plasma concentration of dasatinib achieved in patients with solid tumours (130 nM) [19]. Consistent with our findings, another study which tested dasatinib in a panel of melanoma cell lines, also reported that both Sk-Mel-5 and Sk-Mel-28 are resistant to dasatinib [20]. Interestingly dasatinib treatment resulted in increased proliferation in Sk-Mel-5 and Sk-Mel-28 cells, highlighting the importance of identifying appropriate biomarkers to select patients whose tumours will be growth inhibited by dasatinib and to prevent treatment in cases where dasatinib may promote tumour growth.

SRC is activated by the phosphorylation of tyrosine 418, which regulates proliferation and invasion [2, 21]. However, in melanoma cell lines regardless of their sensitivity to dasatinib, extended exposure to dasatinib inhibited phosphorylation of SRC in all melanoma cell lines tested [20]. Thus, inhibition of SRC alone does not predict sensitivity to inhibition of proliferation by dasatinib in melanoma cells. Although levels of SRC expression are not predictive of response to dasatinib *in vitro*, SRC is expressed in all melanoma cell lines tested and phosphorylation of SRC is inhibited by dasatinib. Therefore, it cannot be overlooked as a potentially important target for dasatinib in melanoma cells. Despite SRC kinase not being a predictive biomarker of response to dasatinib, a number of preclinical studies have shown that targets of the SRC kinase pathway predict response to dasatinib in solid tumours including breast, ovarian and prostate cancer.

By examining a panel of 6-genes which were previously identified as potential biomarkers for dasatinib sensitivity in breast cancer cells [11], we aimed to identify predictive biomarkers for dasatinib in melanoma. Based on previous microarray analysis, 161 genes which were associated with dasatinib sensitivity in 23 breast cancer cell lines were identified. From the list of 161 genes, combined mRNA expression of a 6-gene biomarker panel, comprising of ANXA1, CAV-1, CAV-2, EphA2, IGFBP2 and PTRF, was found to predict response to dasatinib *in vitro* [11]. The 6-gene biomarker panel was also validated in 11 additional breast cancer cell lines and 23 lung cancer cell lines, predicting response to dasatinib in greater than 85 % of cases. These genes are either targets of dasatinib; SRC substrates; or downstream of SRC signalling.

We found that expression of ANXA1, CAV-1, CAV- 2, EphA2, IGFBP2 and PTRF mRNA did not correlate with response to dasatinib in the panel of 8 melanoma cell lines. Interestingly, elevated protein expression of ANXA1, CAV-1 and EphA2, determined by semi-quantitative immuno-blotting, correlated with dasatinib sensitivity in the melanoma cell lines. Expression of CAV-2, IGFBP2 and PTRF did not significantly correlate with dasatinib sensitivity. Previous studies have shown that CAV-1 mRNA expression was elevated in breast and ovarian cell lines that are responsive to dasatinib [11, 12, 15]. Elevated ANXA1 mRNA expression was found in dasatinib sensitive breast and ovarian cell lines [11, 15], whilst EphA2 mRNA was elevated in breast, ovarian and prostate cancer cell lines [11, 14, 15]. We have also previously shown that the level of expression of CAV- 1, CAV-2 and EphA2 protein correlated with sensitivity to dasatinib in breast cancer cell lines [13]. Our study is the first to demonstrate that elevated protein expression of ANXA1, CAV-1 or EphA2 correlates with dasatinib sensitivity in melanoma cells *in vitro.* Statistical analysis of high versus low expression of the biomarkers in the cell line panel suggests that EphA2 alone or in combination with ANXA1 or CAV-1 may have the strongest predictive power. However this observation would need to be confirmed, either in a larger panel of melanoma cell lines or in a cohort of melanoma patients treated with dasatinib.

EphA receptors are targeted by dasatinib [22] and CAV-1 is a downstream target of SRC kinase signalling [23]. SRC kinase has been shown to phosphorylate other members of the annexin family, in particular annexin 2 [24] and we have previously shown that dasatinib treatment results in alterations in the phosphorylation status of ANXA1 and ANXA2 in WM-115 melanoma cells [25]. Interestingly, we have shown a strong association between expression of the 3 proteins in the melanoma cell lines, suggesting that their expression may be co-ordinately regulated in melanoma cells.

We examined expression of the three potential predictive biomarkers (ANXA1, CAV-1 and EphA2) in melanoma specimens by IHC. We detected ANXA1 in 82 % of melanoma samples tested, with no significant difference in the frequency of expression between primary and metastatic melanomas. ANXA1 has not been extensively studied in melanoma, however one study implicated ANXA1 in melanoma metastasis [26, 27].

CAV-1 was expressed in 44 % of the melanoma samples. A previous study of exosomes from melanoma patient plasma found that CAV-1 was expressed at higher levels in melanoma patients compared to healthy volunteers [28]. Consistent with our results, Trimmer *et al* [29] showed that CAV-1 levels were lower in Sk- Mel-28, Sk-Mel-5 and WM-266-4 cells than in the primary melanoma cell line WM-115. They also reported higher expression of CAV-1 in primary (n=30) versus metastatic tumours (n=29) [29]. Jilaveanu *et al* evaluated CAV-1 expression in a cohort of 21 melanoma patients who received dasatinib treatment, in a phase II clinical trial, and observed a trend towards an association between elevated CAV-1 and response to therapy [5].

EphA2 was detected in 74 % of melanoma samples. However, the frequency of EphA2 was higher in primary melanomas (83 %) compared to metastatic tumours (56 %). EphA2 has previously been detected in vertical growth phase cutaneous melanoma samples and strong EphA2 staining was associated with increased melanoma thickness and increased proliferation (Ki67) [30]. Udayakumar *et al* [31] recently showed that EphA2 is frequently overexpressed in a panel of melanoma cell lines, and overexpression of EphA2 in low-expressing cell lines resulted in enhanced growth, colony formation and migration.

Combined expression of SRC kinase, ANXA1, CAV-1 and EphA2 was found in 31 % of tumour samples. Moderate/strong expression of ANXA1, CAV-1 and EphA2 with co-expression of SRC kinase was found in 17 % of melanoma samples. This subpopulation of melanoma patients may be more likely to benefit from dasatinib treatment. Analysis of their clinicopathological features does not indicate any particular differences which would facilitate patient selection (Supplementary Table 8).

Preliminary clinical trials of dasatinib in unselected melanoma patients have yielded disappointing results [8, 32]. However, these trials were conducted in unselected patient populations and it is widely accepted that to achieve maximum effectiveness, targeted therapies require biomarker selected patients. For example, if the HER2- monoclonal antibody trastuzumab had been initially tested in unselected breast cancer patients, the therapeutic impact of trastuzumab may have been missed, as the benefit of trastuzumab is largely restricted to the 20-25% of breast cancer patients whose tumours overexpress HER2 [33].

In conclusion, our results suggest that IHC staining for ANXA1, CAV-1 and/or EphA2 may form the basis of a biomarker panel to select melanoma patients for a biomarker-driven clinical trial of dasatinib, to better define the potential clinical benefit of dasatinib in melanoma treatment.

## METHODS

### Cells and reagents

Lox-IMVI, Malme-3M, M14, Sk-Mel-5, and Sk-Mel-28 were obtained from the Department of Developmental Therapeutics, National Cancer Institute (NCI), HT144 from the American Tissue Culture Centre (ATCC) and WM-115 and WM-266-4 from the European Collection of Cell Cultures (ECACC). Lox-IMVI, Malme-3M, Sk-Mel-5, and Sk-Mel-28 were maintained at 37 °C with 5 % CO_2_ in RPMI medium with 10 % FCS (Cambrex). HT144 was maintained in McCoys 5A (Sigma-Aldrich) with 10 % FCS. WM-115 and WM-266- 4 were maintained in MEM media with 10 % FCS, 2 mM L-glutamine, 1 mM NEAA and 1 mM sodium pyruvate (all Gibco). Stock solutions of dasatinib (10 mM) (Sequoia Research Products) were prepared in dimethyl sulfoxide (Sigma-Aldrich).

### Proliferation assay

Proliferation was measured using an acid phosphatase assay. 1 × 10^3^ cells/well were seeded in 96-well plates, apart from HT144 and Malme-3M which were seeded at 2 × 10^3^ cells/well and incubated overnight at 37°C. Dasatinib was added at the appropriate concentrations and incubated for a further 5 days. Media was removed and the wells were washed once with phosphate buffered saline (PBS). 10 mM paranitrophenol phosphate substrate (Sigma-Aldrich) in 0.1 M sodium acetate buffer with 0.1 % Triton X (Sigma), pH 5.5 was added to each well and incubated at 37 °C for 2 hours. 50 μl of 1 M NaOH was added and the absorbance was read at 405 nM (reference - 620 nM), as previously described [34].

### RNA extraction

RNA extraction was performed using TRI Reagent (Sigma-Aldrich). Cells were grown until 80 % - 90 % confluent in 90 mm petri-dishes. Media was removed and the cells washed twice with PBS. 1 ml of TRI Reagent was added to the plate and then transferred to an eppendorf. 200 μl of chloroform was added, vortexed for 30 seconds, incubated at room temperature for 15 minutes followed by centrifugation at 16,000 x g for 15 minutes at 4 °C. The aqueous layer was removed and 0.5 ml iso-propanol added. Following overnight incubation at −20 °C, the samples were centrifuged at 16,000 x g for 10 minutes at 4 °C. The supernatant was removed and the RNA pellet washed with 1 ml 75 % ethanol. The RNA was air dried, then resuspended in 20 μl of DEPC-treated dH_2_O and stored at −80 °C. RNA concentration and quality was assessed using the NanoDrop (Thermo Scientific).

### Reverse transcriptase reaction

To synthesize cDNA, 2 μl oligo dT_18_ (0.5 μg/ μl) (Sigma-Aldrich), 1 μl DEPC water and 1 μl RNA (1 μg) were heated to 72 °C for 10 min and then cooled to 37 °C. 2 μl 10x MMLV-RT Buffer (Sigma), 0.5 μl RNAsin (40 U/ μl) (Sigma), 1.0 μl 10 mM dNTPs (Sigma), 11 μl DEPC water and 0.5 μl Moloney murine leukaemia virus reverse transcriptase (MMLV-RT) (40,000 U/μl) (Sigma) were added and the reaction incubated at 37 °C for 1 hour. cDNA samples were stored at −20 °C.

### Quantitative real-time PCR (q-RT-PCR)

Taqman® Real Time PCR analysis was performed using the Applied Biosystems Assays On Demand PCR kits (TaqMan® gene expression assays) for ANXA1, CAV- 1, CAV-2, EphA2, PTRF, IGFBP2, on a 7900 fast real-time PCR instrument (Applied Biosystems). Real-Time PCR was performed by adding 22.5 μl of qPCR master mix to the relevant wells of a 96-well PCR plate. qPCR master mix consists of 12.5 μl 2X TaqMan universal PCR mastermix (Applied Biosystems), 1.25 μl 20X gene expression assay mix (Applied Biosystems) and 8.75 μl RNase free water. 2.5 μl of each cDNA sample was added. Biological triplicates of cDNA samples were analyzed in triplicate for measurement of target gene expression and endogenous control (GAPDH).

Expression of each gene was standardized using GAPDH as a reference gene, and relative expression levels for the panel of cell lines were quantified by calculating 2ΔΔ C_T_, where ΔΔC_T_ is the difference in threshold cycle between target and reference genes. Control pooled samples consisting of equal volumes of cDNA of each cell line tested were also prepared. The control pool enabled comparison of the expression of a target gene in a specific cell line relative to the average expression in all the cell lines.

### Preparation of cell extracts for western blotting

500 μL RIPA buffer (Sigma-Aldrich) with 1 X protease inhibitors, 2 mM PMSF and 1 mM sodium orthovanadate (Sigma-Aldrich) was added to cells, incubated on ice for 20 minutes and syringed through an 18 gauge needle. Following centrifugation at 16,000 x g for 5 minutes at 4°C, the resulting lysate was stored at −80°C. Protein quantification was performed using the Bicinchoninic Acid (BCA) assay (Pierce). 30 μg of protein in sample buffer was heated to 95 °C for 5 minutes and proteins were separated on 7.5 or 10 % gels (Lonza). The protein was transferred to Hybond-ECL nitrocellulose membrane (Amersham Biosciences). The membrane was blocked with blocking solution (PBS, 0.1 % Tween, 5 % skimmed milk powder (BioRad)) at room temperature for 1 hour, then incubated overnight at 4 °C with 1 μg/ml primary antibody (mouse anti-EphA2 (Millipore); mouse anti-SRC kinase (Upstate Cell Signalling Solutions); mouse anti-CAV-1 (Cell Signalling Technologies); rabbit anti-PTRF (Santa Cruz Biotechnology); mouse anti-ANXA1 (BD Biosciences); mouse anti-CAV-2 (BD Biosciences); mouse anti-IGFBP2 (Abcam)) in blocking solution. The membrane was washed three times with PBS-Tween, then incubated at room temperature with anti-mouse secondary antibody (Sigma-Aldrich) at 1:1000 dilution or anti-rabbit secondary antibody (Pierce) at 1:3000 dilution in blocking solution for 1 hour. The membrane was washed three times with PBS-Tween followed by one PBS wash. Detection was performed using Luminol (Santa Cruz Biotechnology).

### Immunohistochemistry

The patient group studied comprised 124 malignant melanoma patients diagnosed at St Vincent’s University Hospital (SVUH), Dublin, between 1975 and 2002. Approval to conduct this study was granted by the SVUH Ethics Committee. Representative 4 μm sections of formalin-fixed paraffin-embedded tissue blocks were cut using a microtome, mounted onto poly-l-lysine-coated slides and dried overnight at 37°C.

Deparaffinisation and antigen retrieval was performed using Epitope Retrieval 3-in-1 Solution (pH 6) (DAKO) and the PT Link system (DAKO) for SRC kinase, EphA2 and ANXA1. For CAV-1 deparaffinisation and antigen retrieval was performed using Epitope Retrieval 3-in-1 Solution (pH 9) (DAKO). For epitope retrieval, slides were heated to 97 °C for 20 minutes and then cooled to 65 °C. The slides were then immersed in wash buffer (DAKO). On the Autostainer (DAKO) slides were blocked for 10 minutes with 200 μL HRP Block (DAKO). Cells were washed with 1X wash buffer and 200 μL of antibody was added to the slides for 30 minutes (ANXA1 1: 200; CAV-1 1: 150; EphA2 1: 15; SRC kinase 1: 100). Slides were washed again with 1X wash buffer and incubated with 200 μL Real EndVision (DAKO) for 30 minutes. Slides were washed again with 1X wash buffer and then stained with 200 μL AEC substrate chromagen (DAKO) for 10 minutes and this procedure was repeated twice. A positive control slide was included in each staining run (ANXA1 – tonsil; CAV-1 and SRC kinase – head and neck cancer; EphA2 – metastatic breast cancer). A negative control was also tested for each sample, using antibody diluent without the primary antibody. A blocking peptide was only available for the EphA2 antibody and was used to confirm the specificity of the EphA2 staining (results not shown). All slides were counterstained with haematoxylin (DAKO) for 5 minutes, and rinsed with deionized water, followed by wash buffer. Each slide was mounted with a coverslip using Faramount mounting solution (DAKO) and staining was assessed by consultant pathologist, Professor Susan Kennedy and Alex Eustace.

### Immunohisotchemical scoring

Immunohistochemical staining was evaluated semi-quantitatively, according to the percentage of cells showing specific immunoreactivity and the intensity of this immunoreactivity. Scoring involved evaluation of at least 5 fields of view per slide, by two independent observers (AE and SK). A semi-quantitative measurement was used in which overall positivity of the tumour was assessed and a score of 1+ was given where up to 25% of cells showed positive staining; a score of 2+ was given where ≥ 25% but < 50% of cells showed positive staining; a score of 3+, where ≥ 50% of cells showed positive staining. For assessment of intensity of staining, the intensity of immunoreactivity was scored as 1 (weak), 2 (moderate), or 3 (strong) as outlined in table 1.

### Statistical analysis

Statistical analyses were performed using StatView 5.0.1 (SAS Institute). Comparisons between expression of the 6-panel gene markers at both the protein and RNA levels, in dasatinib sensitive and resistant cell lines, were analysed using the Student’s t-test. Pearson correlation coefficient analysis was used to examine the correlation between mRNA and protein expression of the 6-gene panel in the panel of melanoma cell lines. Chi-squared and Kruskal-Wallis tests were used to evaluate associations between protein expression and patient clinicopathological parameters.

## SUPPLEMENTARY FIGURES AND TABLES


