# Supplementary material for: Predictive biomarkers for dasatinib treatment in melanoma
Source: Oncoscience. 2014 Mar 12;1(2):158–66. doi: 10.18632/oncoscience.20 (PMC4278284; doi:10.18632/oncoscience.20)
Supplement: Supplementary file 1 [file oncoscience-01-0158-s001.pdf]

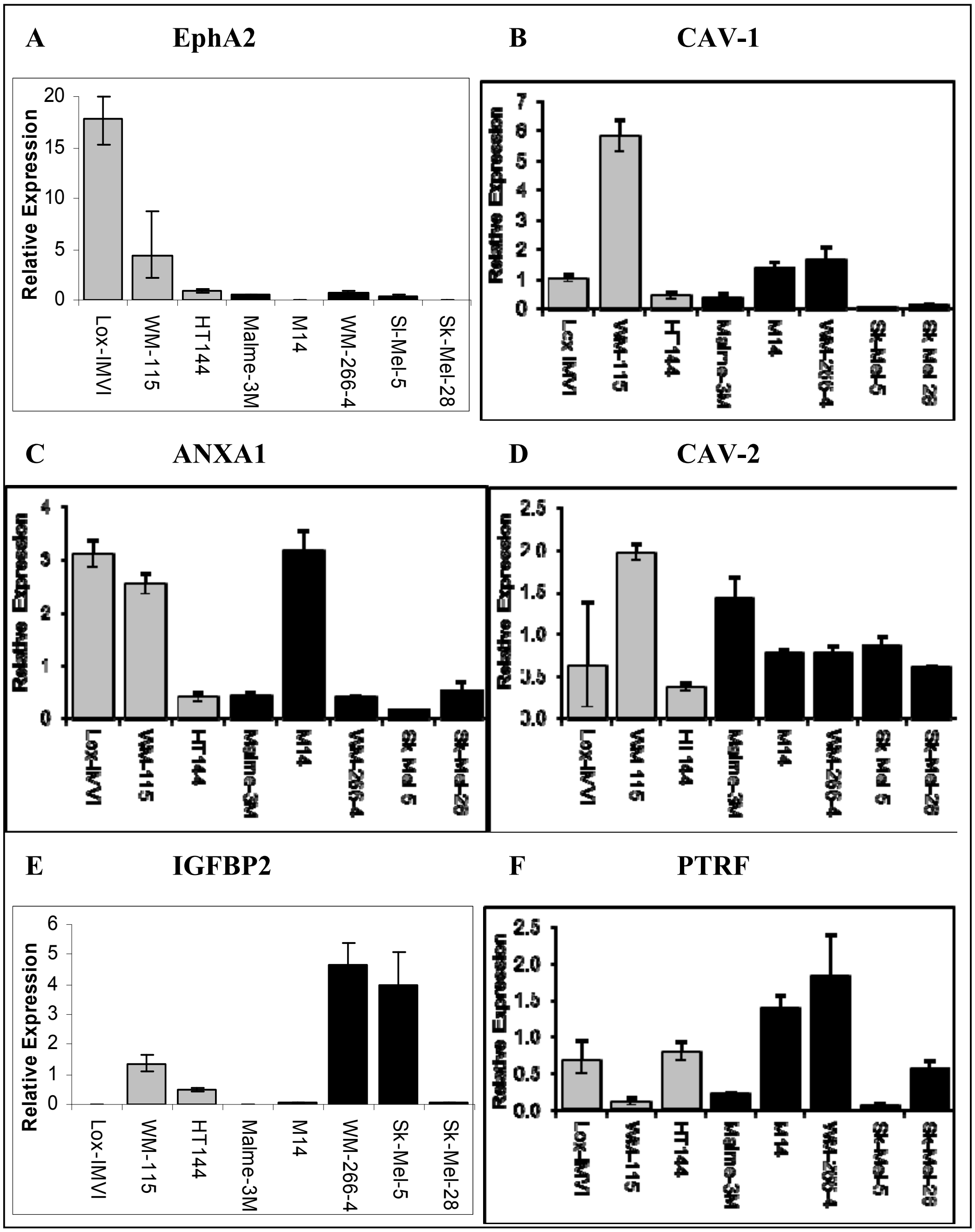

Supplementary figure 1: Relative expression levels of A) EphA2, B) CAV-1, C) ANXA1, D) CAV-2, E) IGFBP2 and F) PTRF mRNA measured by qRT-PCR. Relative expression is measured compared to the control mix (a combination of equal amounts of mRNA from each cell line tested). Black bars represent dasatinib resistant cell lines; grey bars represent dasatinib sensitive cell lines. A result less than 1 indicates lower expression than the control whilst a result of greater than 1 indicates elevated expression relative to the control. Error bars represent standard deviations for triplicate qRT-PCR results.

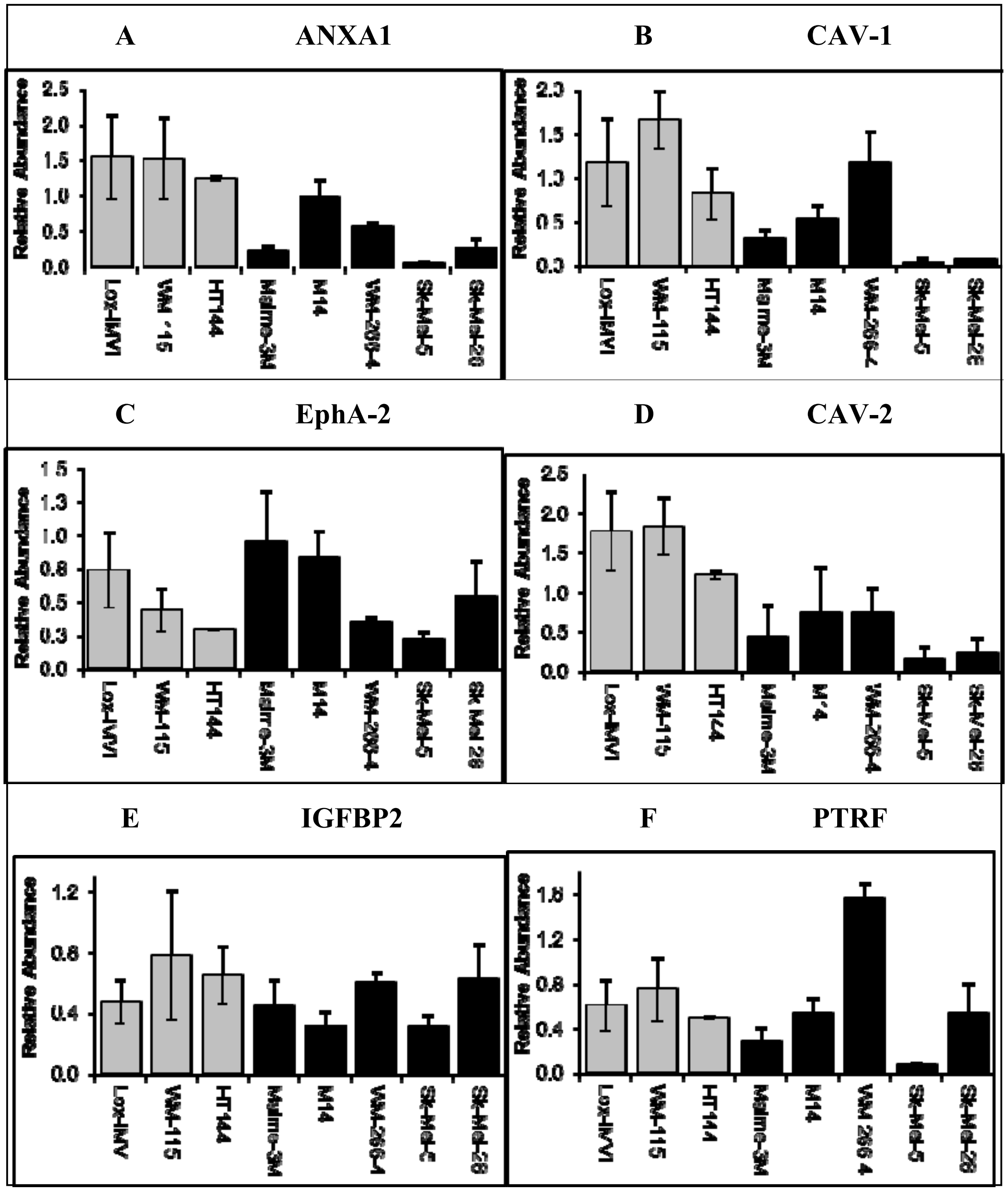

Supplementary figure 2: Relative expression levels of A) ANXA1, B) CAV-1, C) EphA2, D) CAV-2, E) IGFBP2 and F) PTRF protein levels measured by western blotting. Relative expression was measured by densitometry measurement of band intensities compared to the α-tubulin endogenous control. Black bars represent dasatinib resistant cell lines; grey samples represent dasatinib sensitive cell lines. Error bars represent standard deviations for triplicate independent experiments.

Supplementary Table 1: Correlation between each of the individual markers and combinations of the markers, and sensitivity to dasatinib. Each cell line was categorized as a high or low expressor for the individual markers using the median level of expression as a cut-off.

	**Chi-Squared test, p value**
ANXA1	0.0285
CAV-1	0.0285
EphA2	0.0047
ANXA1 + CAV-1	0.0183
ANXA1 + EphA2	0.0907
CAV-1 + EphA2	0.0907
ANXA1 + CAV-1 + EphA2	0.0183

Supplementary Table 2: Pearson Rank correlation between mRNA and protein levels in ANXA1, CAV-1, EphA2, CAV-2, IGFBP2 and PTRF.

**Biomarker**	**mRNA vs protein levels**	
ANXA1	p = 0.115	r = 0.619
CAV-1	p = 0.002	r = 0.881
EphA2	p = 0.058	r = 0.714
CAV-2	p = 0.619	r = 0.214
IGFBP2	p = 0.665	r = 0.190
PTRF	p = 0.229	r = 0.429

Supplementary table 3: Clinicopathological characteristics of patient samples used for immunohistochemical analysis of ANXA1, CAV-1 and EphA2 expression.

Characteristic	Primary melanoma n = 82	Metastatic melanoma n = 42
**Age (years)**		
≤ 60	26 (32 %)	16 (38 %)
> 60	56 (68 %)	26 (62 %)
**Gender**		
Male	25 (30 %)	21 (50 %)
Female	57 (70 %)	21 (50 %)
**Breslow thickness (mm)**		
Unknown	13 (21 %)	
< 1	27 (39 %)	
1 – 1.9	10 (14 %)	
2 – 3.9	12 (17 %)	
> 4	20 (29 %)	
Grouping 0 - 2	37 (54 %)	
Grouping > 2	32 (46 %)	
**Clarke's Level**		
Unknown	19 (23 %)	
I	0 (0 %)	
II	8 (13 %)	
III	28 (44 %)	
IV	20 (32 %)	
V	7 (11 %)	
**Lymph node status**		
Unknown	55 (67 %)	
Positive	6 (22 %)	
Negative	21 (78 %)	

Supplementary table 4: Correlation between clinico-pathological factors and number of SRC positive tumours; percentage of tumour cells positive for SRC expression (0 = 0%; 1 = <25 %; 2 = >25 % - <50 %; 3 = ≥50 %); and intensity of SRC staining in tumour cells in primary melanoma specimens (0 = negative; 1 = weak; 2 = moderate; 3 = strong). '*' indicates a P value of < 0.05 as determined by Chi-Squared or Kruskal-Wallis test.

**SRC Kinase**	**Characteristic**	**SRC positive**	**% tumour cells positive for SRC**			**Intensity of staining in tumour cells**			
			**0**	**≤ 50**	**> 50**	**0**	**1**	**2**	**3**
**Age**	**< 60 years**	25	2/27	11/27	14/27	2/27	6/27	12/27	7/27
	**≥ 60 years**	41	14/55	19/55	22/55	14/55	9/55	14/55	18/55
**Gender**	**Male**	22	3/25	8/25	14/25	3/25	4/25	8/25	10/25
	**Female**	44	13/57	22/57	22/57	13/57	11/57	18/57	15/57
**SLN Status**	**Positive**	4	2/6	3/6	1/6	2/6	3/6	0/6	1/6
	**Negative**	18	3/21	7/21	11/21	3/21	3/21	6/21	9/21
**Clark's level**	**II**	8	0/8	4/8	4/8	0/8	3/8	2/8	3/8
	**III**	23	5/28	8/28	15/28	5/28	1/28	14/28	8/28
	**IV**	17	3/20	9/20	8/20	3/20	5/20	4/20	8/20
	**V**	6	1/7	2/7	4/7	1/7	2/7	1/7	3/7
**Grouped Clarke's level**	**< 4**	31	5/36	12/36	19/36	5/36	4/36	16/36	11/36*
	**4, 5**	23	4/27	11/27	12/27	4/27	7/27	5/27	11/27
**Breslow thickness**		57	12/69	26/69	31/69	12/69	14/69	21/69	22/69
**Breslow thickness (mm)**	**< 1**	22	5/27	10/27	12/27	5/27	7/27	9/27	6/27
	**1.0 – 1.9**	7	3/10	3/10	4/10	3/10	0/10	3/10	4/10
	**2.0 – 3.9**	10	2/12	4/12	6/12	2/12	1/12	6/12	3/12
	**> 4**	18	2/20	9/20	9/20	2/20	6/20	3/20	9/20
**Breslow grouped**	**≤ 2**	30	8/38	14/38	16/38	8/38	7/38	13/38	10/38
	**> 2**	27	4/31	12/31	15/31	4/31	7/31	8/31	12/31

Supplementary table 5: Correlation between clinico-pathological factors and number of ANXA1 positive tumours; percentage of tumour cells positive for ANXA1 expression (0 = 0%; 1 = <25 %; 2 = >25 % - <50 %; 3 = ≥50 %); and intensity of ANXA1 staining in tumours in primary melanoma specimens (0 = negative; 1 = weak; 2 = moderate; 3 = strong). '*' indicates a P value of < 0.05 as determined by Chi-Squared or Kruskal-Wallis test.

**ANXA1**		**ANXA1 Positive**	**% of tumor cells positive for ANXA1**				**Intensity of ANXA1 staining**			
			0	1	2	3	0	1	2	3
**Age**	**< 60 years**	22	3/25	7/25	5/25	10/25	3/25	3/25	9/25	10/25
	**≥ 60 years**	45	11/56	18/56	9/56	18/56	11/56	2/56	16/56	27/56
**Gender**	**Male**	22	2/24	7/24	4/24	11/24	2/24	3/24	7/24	12/24
	**Female**	45	12/57	18/57	10/57	17/57	12/57	2/57	18/57	25/57
**SLN status**	**Positive**	5	1/6	2/6	0/6	3/6	1/6	0/6	2/6	3/6
	**Negative**	17	4/21	9/21	3/21	5/21	4/21	2/21	8/21	7/21
**Clarke's level**	**2**	6	1/7	1/7	2/7	3/7	1/7	2/7	2/7	2/7
	**3**	26	3/29	9/29	7/29	10/29	3/29	1/29	10/29	15/29
	**4**	15	5/20	3/20	5/20	7/20	5/20	0/20	5/20	10/20
	**5**	5	1/6	2/6	0/6	3/6	1/6	0/6	1/6	4/6
**Grouped Clarke's level**	**≤ 4**	32	4/36	10/36	9/36	13/36	4/36	3/36	12/36	17/36
	**4,5**	20	6/26	5/26	5/26	10/26	6/26	0/26	6/26	14/26
**Breslow thickness**		56	11/67	18/67	14/67	24/67	11/67	3/67	21/67	32/67 *
**Breslow thickness (mm)**	**< 1**	24	3/27	9/27	9/27	6/27	3/27	2/27	12/27	10/27
	**1.0 – 1.9**	7	3/10	3/10	1/10	4/10	3/10	0/10	4/10	3/10
	**2.0 – 3.9**	10	1/11	2/11	2/11	5/11	1/11	1/11	1/11	8/11
	**> 4**	15	4/19	4/19	2/19	9/19	4/19	0/19	4/19	11/19
**Breslow grouped**	**≤ 2**	32	6/38	12/38	10/38	10/38	6/38	2/38	16/38	14/38
	**> 2**	24	5/29	6/29	4/29	14/29	5/29	1/29	5/29	18/29

Supplementary table 6: Correlation between clinico-pathological factors and number of CAV-1 positive tumours; percentage of tumour cells positive for CAV-1 expression (0 = 0%; 1 = <25 %; 2 = >25 % - <50 %; 3 = ≥50 %); and intensity of CAV-1 staining in tumour cells in primary melanoma specimens (0 = negative; 1 = weak; 2 = moderate; 3 = strong). '*' indicates a P value of < 0.05 as determined by Chi-Squared or Kruskal-Wallis test.

**Caveolin-1**		**CAV-1 Positive**	**% of tumor cells positive for CAV-1**				**Intensity of staining in tumor cells**			
			0	1	2	3	0	1	2	3
**Age**	**< 60 years**	19 *	8/27	12/27	4/27	3/27*	8/27	7/27	7/27	5/27*
	**≥ 60 years**	20	35/55	8/55	10/55	2/55	35/55	3/55	11/55	6/55
**Gender**	**Female**	24	34/58	10/58	9/58	5/55*	34/58	6/58	10/58	8/58
	**Male**	15	9/24	10/24	5/24	0/24	9/24	4/24	8/24	3/24
**SLN status**	**Positive**	4	2/6	1/6	2/6	1/6	2/6	0/6	2/6	2/6
	**Negative**	11	10/21	5/21	4/21	2/21	10/21	3/21	3/21	5/21
**Clarkes level**	**2**	7	1/8	5/8	1/8	1/8	1/8	3/8	3/8	1/8
	**3**	13	16/29	6/29	4/29	3/29	16/29	4/29	4/29	5/29
	**4**	10	9/19	4/19	5/19	1/19	9/19	2/19	5/19	3/19
	**5**	5	2/7	1/7	4/7	0/7	2/7	0/7	3/7	2/7
**Grouped Clarke's level**	**≤ 4**	20	17/37	11/37	5/37	4/37	17/37	7/37	7/37	6/37
	**4,5**	15	11/26	5/26	9/26	1/26	11/26	2/26	8/26	5/26
**Breslow thickness**		35	34/69	16/69	14/69	5/69	34/69	9/69	15/69	11/69
**Breslow thicknes (mm)**	**< 1**	17	11/28	9/28	5/28	3/28	10/28	5/28	8/28	5/28
	**1.0 – 1.9**	3	6/9	1/9	2/9	0/9	6/9	1/9	1/9	1/9
	**2.0 – 3.9**	3	9/12	2/12	0/12	1/12	9/12	1/12	1/12	1/12
	**> 4**	12	8/20	4/20	7/20	1/20	8/20	2/20	6/20	4/20
**Breslow grouped**	**≤ 2**	21	17/38	11/38	7/38	3/38	17/38	7/38	8/38	6/38
	**> 2**	14	17/31	5/31	7/31	2/31	17/31	2/31	7/31	5/31

Supplementary table 7: Correlation between clinico-pathological factors and number of EphA2 positive tumours; percentage of tumour cells positive for EphA2 expression (0 = 0%; 1 = <25 %; 2 = >25 % - <50 %; 3 = ≥50 %); and intensity of EphA2 staining in tumour cells in primary melanoma specimens (0 = negative; 1 = weak; 2 = moderate; 3 = strong). '*' indicates a P value of < 0.05 as determined by Chi-Squared or Kruskal-Wallis test.

**EphA2**		**EphA2 Positive**	**% of tumor cells positive for EphA2**				**Intensity of staining in tumor cells**			
			0	1	2	3	0	1	2	3
**Age**	**< 60 years**	24	3/27	5/27	4/27	15/27	3/27	2/27	10/27	12/27
	**≥ 60 years**	44	11/55	6/55	16/55	22/55	11/55	12/55	20/55	12/55
**Gender**	**Female**	48	9/57	7/57	17/57	24/57	9/57	10/57	25/57	13/57
	**Male**	20	5/25	4/25	3/25	13/25	5/25	4/25	5/25	11/25
**SLN status**	**Positive**	5	1/6	1/6	0/6	4/6	1/6	1/6	1/6	3/6
	**Negative**	17	4/21	4/21	4/21	9/21	4/21	3/21	10/21	4/21
**Clarkes level**	**2**	8	0/8	1/8	2/8	5/8	0/8	1/8	4/8	3/8
	**3**	23	4/27	2/27	9/27	12/27	4/27	4/27	9/27	10/27
	**4**	17	3/20	1/20	4/20	12/20	3/20	5/20	6/20	6/20
	**5**	7	0/7	1/7	3/7	3/7	0/7	3/7	3/7	1/7
**Grouped Clarke's level**	**Level ≤ 4**	31	4/35	3/35	11/35	17/35	4/35	5/35	13/35	13/35
	**Level 4,5**	24	3/27	2/27	7/27	15/27	3/27	8/27	9/27	7/27
**Breslow thickness**		58	10/68	6/68	19/68	33/68	10/68	13/68	23/68	22/68
**Breslow thickness (mm)**	**< 1**	21	5/26	1/26	9/26	11/26	5/26	2/26	11/26	8/26
	**1.0 – 1.9**	8	2/10	0/10	3/10	5/10	2/10	2/10	5/10	1/10
	**2.0 – 3.9**	12	0/12	4/12	2/12	6/12	0/12	3/12	3/12	6/12
	**> 4**	17	3/20	1/20	5/20	11/20	3/20	6/20	4/20	7/20
**Breslow grouped**	**≤ 2**	30	7/37	2/37	12/37	16/37	7/37	5/37	16/37	9/37
	**> 2**	28	3/31	4/31	7/31	17/31	3/31	8/31	7/31	13/31
